# The clinical and translational research activities at the INT – IRCCS “Fondazione Pascale” cancer center (Naples, Italy) during the COVID-19 pandemic

**DOI:** 10.1186/s13027-020-00330-7

**Published:** 2020-11-23

**Authors:** Franco M. Buonaguro, Gerardo Botti, Paolo Antonio Ascierto, Sandro Pignata, Franco Ionna, Paolo Delrio, Antonella Petrillo, Ernesta Cavalcanti, Maurizio Di Bonito, Sisto Perdonà, Michelino De Laurentiis, Francesco Fiore, Raffaele Palaia, Francesco Izzo, Stefania D’Auria, Virginia Rossi, Simona Menegozzo, Mauro Piccirillo, Egidio Celentano, Arturo Cuomo, Nicola Normanno, Maria Lina Tornesello, Rocco Saviano, Daniela Barberio, Luigi Buonaguro, Giovanni Giannoni, Paolo Muto, Leonardo Miscio, Attilio A. M. Bianchi, and the INT-Pascale COVID-19 Crisis Unit, Marco Cascella, Paolo Ciavolino, Marco Correra, Rossella Di Franco, Gaetano Di Pietro, Pierluigi Franco, Giovanni Grizzuti, Francesco Labonia, Sergio Russo, Giovanni Salzano

**Affiliations:** Istituto Nazionale Tumori IRCCS Fondazione Pascale, Naples, Italy

**Keywords:** COVID-19, SARS-CoV-2, ARDS, Cancer center, Italy, Pascale, IL-6, Tocilizumab

## Abstract

COVID-19 pandemic following the outbreak in China and Western Europe, where it finally lost the momentum, is now devastating North and South America. It has not been identified the reason and the molecular mechanisms of the two different patterns of the pulmonary host responses to the virus from a minimal disease in young subjects to a severe distress syndrome (ARDS) in older subjects, particularly those with previous chronic diseases (including diabetes) and cancer. The Management of the Istituto Nazionale Tumori - IRCCS “Fondazione Pascale” in Naples (INT-Pascale), along with all Health professionals decided not to interrupt the treatment of those hospitalized and to continue, even if after a careful triage in order not to allow SARS-CoV-2 positive subjects to access, to take care of cancer patients with serious conditions. Although very few (*n* = 3) patients developed a symptomatic COVID-19 and required the transfer to a COVID-19 area of the Institute, no patients died during the hospitalization and completed their oncology treatment. Besides monitoring of the patients, all employees of the Institute (physicians, nurses, researchers, lawyers, accountants, gatekeepers, guardians, janitors) have been tested for a possible exposure. Personnel identified as positive, has been promptly subjected to home quarantine and subdued to health surveillance. One severe case of respiratory distress has been reported in a positive employees and one death of a family member. Further steps to home monitoring of COVID-19 clinical course have been taken with the development of remote Wi-Fi connected digital devices for the detection of early signs of respiratory distress, including heart rate and oxygen saturation.

In conclusion cancer care has been performed and continued safely also during COVID-19 pandemic and further remote home strategies are in progress to ensure the appropriate monitoring of cancer patients.

## Introduction

**Fig. 1:**
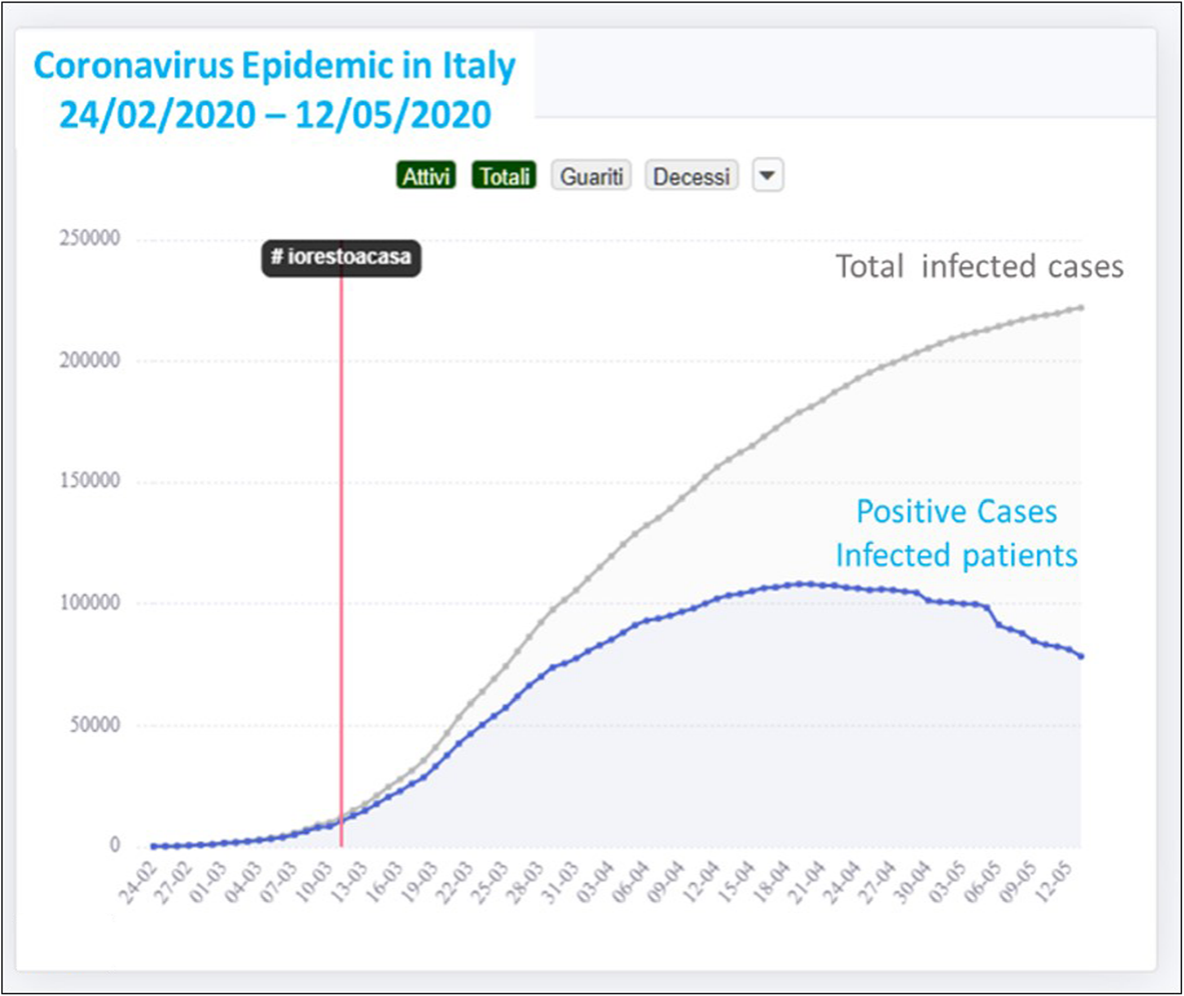
Incidence of the 2020 SARS-CoV-2 Pandemic in Italy in the period February 24–May 12

**Fig. 2:**
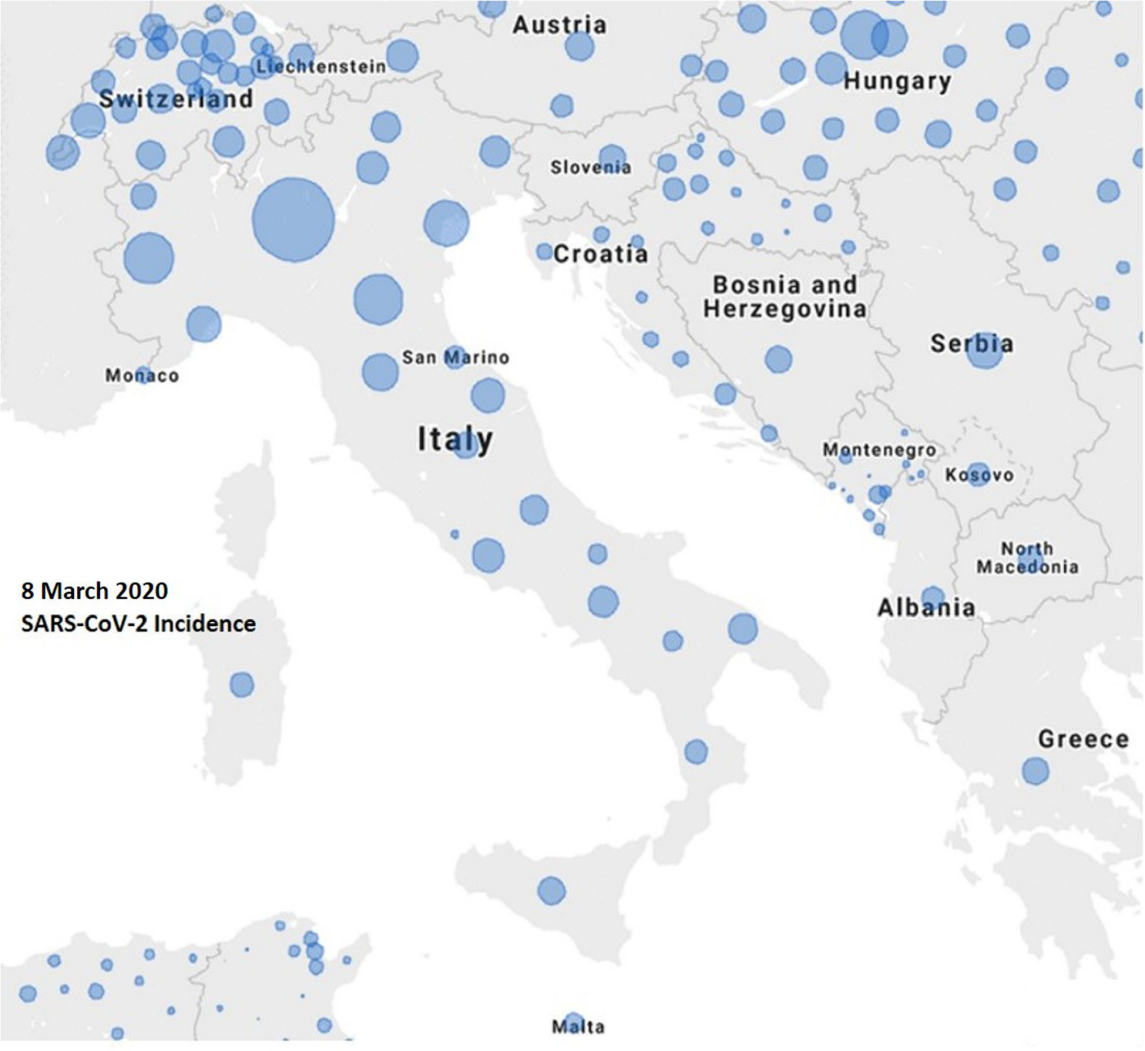
Incidence on March 8 of the 2020 SARS-CoV-2 Pandemic in Italy, articulated by Regions

As of June 1, 2020, coronavirus disease 2019 (COVID-19) has been confirmed in ~ 6,000,000 people worldwide, carrying a mortality of approximately 6.8% [1], compared with a mortality rate of less than 1% from influenza. Italy (especially Northern Italy) has been badly it by the epidemic in the March–April period (Figs. 1 and 2). The epidemic in January was mainly restricted to China and few cases in South-East Asia (Thailand, Malaysia, Singapore, Taiwan, Japan and South Korea) and in Europe (France and Germany) [2]. Almost a month later, on February 16th, the first COVID-19 was identified in Italy at Codogno Hospital (Lodi) (https://www.repubblica.it/cronaca/2020/02/22/news/cina_coronavirus_italia_virus_wuhan_influenza_codogno_lombardia_veneto_adriano_trevisan-249215365/). In few days two outbreak sites were identified 15 cases in Lodi (Lombardy) and 4 cases in Vo’ (Veneto) and on Feb 21st the first Italian COVID-19 died in Padua [3]. On March 8th the Italian epidemic became the second largest in the world behind Korea (outside of mainland China) and the largest in the Western countries in terms of COVID-19 confirmed cases with 6387 cases, including 366 deaths. The majority of these cases were located in northern regions, with Lombardy and Emilia Romagna reporting a combined 5369 cases (84%). The Italian government, consequently took a number of steps in an attempt to limit the spread of SARS-CoV-2 and over the weekend, cordoned a region containing almost a quarter of all Italian citizens The measures did not permit the travel in or out of Lombardy and other surrounding towns, except for proven work or emergency-related reasons [4].

On March 10th in Italy, Europe’s worst affected country, total confirmed cases jumped to 9172 with 463 deaths, making it the 1st largest outbreak outside of mainland China. The Prime Minister expanded movement restrictions to the entire country. The decision came after a spike in deaths from COVID-19. The measures include cancellation of all public gatherings, restriction of movement throughout the country, and an extension of school and university closures through the following months [5]. On March 12th the Italian Ministry of Health reported a total of 12,839 cases and 1016 deaths. *The New York Times* reported that Italy’s health system had become rapidly overwhelmed in just 3 weeks, leading to severely limited availability for ventilators and necessitating difficult decisions regarding how ration medical care among patients. The rapid spread and increase in the number of severe cases prompted Italy to enforce radical social distancing measures and to strongly encourage other countries to rapidly implement these measures as well [6]. On March 19th Italy had overtaken China with respect to the number of reported COVID-19 deaths, reporting a total of 3405 deaths compared to 3248 reported deaths in China; however, China’s 80,967 cases remained the highest national total, and Italy was second with 33,190 [7]. The speed of the epidemic and the associated damages were evident from the first week of March (Fig. 1) and the Hospital Management (led by the Director General, Dr. Attilio Bianchi), concerned about the risk of frail patients present in the Cancer Institute, for their age and their comorbidities, has begun to develop possible COVID-19 therapeutic strategies in addition to establishing the COVID- 19 Crisis unit, coordinated by the Medical Director. The therapeutic strategy based on inhibition of IL-6R has been previously published [8–10] and will not be furthermore elaborate in this article, which will be focused on the strategies adopted to prevent COVID-19 spreading among the susceptible cancer patients and their care givers, as well as the Cancer Institute Health workers. Moreover, the IRCCS Pascale under the coordination of the Scientific Director participated to COVID-19 Research Programs launched by the Campania Regional Government, as well as by the Italian Ministry of Health.

The mission of a comprehensive Cancer Center in a country with implementation of an advanced welfare and universal health system (adopted in Italy since 1978), besides implementing translational research and clinical trials with the most innovative treatment, must include cancer prevention research and territorial Regional coordination of cancer early diagnosis and follow-up programs (in Campania the Oncological Network is coordinated by the INT-Pascale since 2017). The organization in Departments dedicated to specific cancers (i.e. breast and head and neck cancers) and multidisciplinary and integrated teams (GOM, multispecialty oncological groups, made by oncologists, surgeons, radiotherapist, radiologists, pathologists, biologists and virologists, depending on the cancer type and etiology) accelerated the involvement of the whole Institute also in the COVID-19 emergency. In a contest where research, patient clinical care and health management within a global multidisciplinary philosophy are contributing to improving health programs and outcomes, the COVID-19 pandemic had to be tackled according to an unprecedented knowledge management and innovation approach [11, 12], which allows research, clinical science and management to stimulate each other to achieve an institutional synergistic collective intelligence [13] and even a global collective intelligence [14].

### The COVID-19 activities at the INT-Pascale

The major areas of translational research pursued at the Pascale Institute have been aimed at identifying the mechanisms underlying the pathogenesis of COVID-19 to improve diagnostic and therapeutic approaches to this disease. In particular, the following activities have been focused on:

1. The construction of a regional database in which to record all the COVID-19 cases in order to analyze the correlation between biomarkers and clinical-pathological characteristics;
2. The start of a monitoring activity for the spread of the epidemic among health workers;
3. Sequencing of the SARS-CoV-2 virus to monitor possible evolution;
4. Characterization of the immune response to SARS-CoV-2;
5. The study of the role of innate immunity and the ACE2 receptor in SARS-CoV-2 infection;
6. The characterization of mediators of the inflammation in patients with COVID-19.

The Institute decision to ensure continuous commitment to and treatment of cancer patients determined the reorganization of most clinical oncology activities in the medical and the surgical oncology wards as well as in diagnostic units and the sectors dealing mainly with not hospitalized patients (i.e. radiotherapy and interventional radiology).

### COVID-19 impact on medical oncology

The INT-Pascale has a major focus on cancer molecular characterization, biomarkers identification and implementation of tailored precision medicine with the backing of innovative therapeutic strategies. In line with this strategy, the Institute and their clinical units are members of several networks contributing and coordinating several national and international European clinical trials for different type of cancers. Most conventional and palliative treatments are left to smaller oncology units distributed in the Campania Region, which are part of the regional oncology network, coordinated by the Pascale Cancer Institute.

In the 50-day period (March 10th-April 30th) the COVID-19 pandemic heavily influenced the conduction of clinical trials at the Pascale Institute, as in many other European countries, by the excess load in Intensive and sub-intensive care units (ICU) of COVID-19 patients affected by severe respiratory syndrome (ARDS). Moreover, the clinical trial conduction has been severely hampered by the national lockdown, with limitation of patients and caregivers free circulation, and even more by the severe restriction imposed by the Campania Governor in order to achieve social distancing by the home isolation for the all population.

Our institute, however, maintained a strong commitment to reduce the impact of COVID-19 on ongoing clinical trials and, in agreement with regulatory agencies, pursued their pivotal principals: assuring the safety of trial participants; − maintaining compliance with good clinical practice (GCP); − minimizing risks to trial integrity and quality of data. Although with varying difficulties, most of the Pascale clinical research team, in line with the national regulatory agency AIFA and the EMA, adopted the following general rules:

1. Continuation of multicenter clinical trials coordinated by the Pascale Institute.

In accordance with AIFA, the following recommendations were issued to continue clinical trials in centers where possible and to prepare a specific risk plan. In particular:

- give instructions to the sites on how to proceed for enrollment, investigational medical product (IMP) distribution and visits;
- check the IMP stock at the sites;
- contact the couriers to guarantee the delivery of IMP;
- continue to include patients in ongoing trials with open recruitment at internal and external sites if they can guarantee the standard health care of the patient in case of any complication related to the disease, the IMP or the COVID-19 infection, as well as the compliance with the protocol

2. Participation of Pascale Units to protocols coordinated by other groups or Pharma

Patients, who already signed the Informed consent form should be included in the trial as far as the patient fulfills all the inclusion criteria and none of the exclusion criteria. New patients can be also considered.

IMP should not be stopped unless there is an issue with access to drug or concern regarding the possibility of providing the optimal health care to the patient. In the latter it can be considered a return to standard treatment.

Management of COVID-19 positive patients enrolled in a clinical trial is expected to follow the regular protocols. In symptomatic patients, IMP must be postponed until the patient’s recovery. In the case of asymptomatic COVID-19 positive patients, it is reasonable to delay IMP administration until the patient will become COVID-19 negative or 14 days had passed without symptoms.

For oral medications, taking into account patient mobility limitations and with the aim of reducing potential patient exposure to COVID-19, one could choose to dispense and ship the oral IMP to the patient by courier or mail. In this case, a clinical evaluation is mandatory before continuing the oral IMP and can be carried out by telephone or video consultation and documented in the medical record.

The communication of blood results can be done by teleconsultation. When the patient is unable to visit the hospital, blood tests at another facility close to the patient should be considered and the researcher informed via teleconsultation.

It is also recommended to maintain the recruitment of PRO questionnaires during this period.

Investigators are strongly encouraged to contact the Sponsor for information on any circumstances that may impact the trial management at sites during the COVID-19 pandemic in order to set up a mitigation plan. This is particularly relevant when considering stopping patient recruitment or stopping trial treatment. The investigator and the sponsor are advised to find the best solution for the patient, including the administration delay of IMP or even the patient transfer to another site.

In order to limit the spread of COVID 19 and guarantee the continuity of oncological treatments, some suggestions were provided by a board of Italian oncologists belonging to the main scientific societies (AIOM, CIPOMO and COMU) [15].

According to these advices, it was suggested to carefully balance risks and benefits of oncological therapies and to prioritize treatment during this pandemia. In clinical practice, one possible strategy could be to delay, as far as possible, adjuvant treatments or to prefer schedule with longer interval (this is what it was also recommended by ESMO for melanoma) [16].

Telemedicine could be used in patients in immunotherapy, or in treatment with weekly chemotherapy, or in maintenance therapy, performing blood tests in laboratory near to patient’s home, and with medical assessment by phone. This approach could also be used in patients in follow up with no evidence of recurrence at radiological exams telematically checked.

If it is not necessary, it should be avoided the access of caregivers at scheduled therapy visit and in day hospital area, where treatment is administered. It is deeply suggested to forbid access of patient with respiratory syndrome or with fever who have not undergone any previous triage; in these patients strongly we recommend to perform a nasal swab to ascertain the possibility of a COVID-19.

Another issue is differential diagnosis in patients with suspicious respiratory symptoms undergoing immunotherapy or other drugs that can cause as adverse event interstitial pneumonia, or in patients on treatment with BRAFi/MEK inhibitors affected by fever that does not resolve with treatment interruption. In these cases, we should require a COVID test.

Considering data available on cancer patients and COVID-19 [17, 18], oncological decisions during this pandemia should be taken prudently and according to the latest evidences and scientific recommendations.

### COVID-19 impact on surgical oncology

The major impact of COVID-19 within the surgery oncology activities is mainly on the respiratory and gastro-digestive tract for the direct infection of lining epithelial and organs by the SARS-CoV-2. Specific epithelial cells of both districts (i.e. epithelial cells of lung alveoli and bile ducts) have on the surface glycoprotein ACE2 molecules, which represent the binding site (receptor) for the viral Spike molecules. The Angiotensin-converting enzyme 2 (ACE2) is an enzyme attached to the outer surface (cell membranes) of cells in the lungs, arteries, heart, kidney, and intestines. ACE2 lowers blood pressure by catalysing the hydrolysis of angiotensin II (a vasoconstrictor peptide) into angiotensin (1–7) (a vasodilator). ACE2 counters the activity of the related angiotensin-converting enzyme (ACE) by reducing the amount of angiotensin-II and increasing Ang (1–7). For such reason specific precautions must be taken during the surgery as well as during the post-surgery curettage/ bandaging for outpatient treatment, in particular of tracheostomy or colostomy patients.

### Surgical oncology of the head and neck (H&N) district during COVID-19 pandemic

The main mechanism of transmission of COVID-19 is through the air, followed by, to a lesser extent, direct or close contact and finally by the fecal-oral route. Transmission is generally achieved through contact with a symptomatic case, but is also possible from an asymptomatic infected person. For this reason, healthcare workers who come into contact with the upper aero-digestive tract during diagnostic and therapeutic procedures - such as otolaryngologists, head and neck surgeons, oral and maxillofacial surgeons and dentists - are particularly at risk because of the rapid spread of this disease through respiratory droplets [19, 20].

Moreover, all head and neck inspections are considered high-risk procedures and, given the risk of contagion from completely asymptomatic patients during this pandemic, every patient must be considered a possible cause of transmission. Therefore, it is mandatory to adopt the right precautions during patients’ clinical inspection, and, subsequently, during hospitalization/intra-operative/post-surgical time (Table 1).

**Table 1:** Table 1

Head and Neck Guidelines	
1. Select the patients carefully. If the tracheostomy is assessed as difficult because of anatomy, history of comorbidities, or other factors, consider postponing the procedure.	
2. Considerations may be given to percutaneous dilatational tracheotomy if the patient’s anatomy and proceduralist expertise allows it to be done safely with minimal or no bronchoscopy, endotracheal suctioning, and disruption of the ventilator circuit.	
3. Provide adequate sedation including paralysis to eliminate the risk of coughing during the procedure. Ventilation should be paused (apnea at end expiration when the trachea is entered and any time the ventilation circuit is disconnected.	
4. Choose a non-fenestrated, cuffed. Tracheostomy tube on the smaller side to make the tracheotomy hole smaller overall (Shiley size 6 to 8.5 for both men and women are adequate). Keep the cuff inflated to limit the spread of virus through the upper airway.	
5. Perform tracheotomy suctioning using a closed suction system with a viral filter.	
6. Use a heat moisture exchanger device instead of a tracheotomy collar during weaning to prevent virus spread or reinfection of patients.	
7. Avoid changing the tracheotomy tube until viral load is as low as possible.	

Based on the experience gained in Wuhan, China, and Northern Italy, it was evident that N95 masks were not sufficient to control the dissemination of the disease. Indeed, it was not until PAPR (Powered Air Respiratory Protection) was introduced that the transmission of the virus was controlled among medical personnel [20].

During ENT examination with flexible laryngoscope, in order to reduce nausea and irrigated cough, the smallest possible laryngoscope diameter was employed associated to the use of anesthetic gel, that replaced local anesthetic spray [21]. For each patient a disposable device, that covers the endoscope, was used. The same procedure was performed during nasal fibroendoscopy, to ensure adequate surface anesthesia in the way of reducing sneeze reflex. All endoscopic procedures were performed safely both for operators and patients.

High-risk operations were performed with appropriate Personal Protective Equipment (PPE) worn by the healthcare personnel. Considering the high viral titles in nasal mucosal, oral, pharyngeal, and pulmonary secretions, every intervention that involves these surfaces/fluids is at high-risk to the entire operating room personnel. This includes the use of powered devices (eg, drills, microdebriders, saws) or ultrasonic shears, such as the Harmonic scalpel (Ethicon) or Thunderbeat scalpel (Olympus).

In every surgical procedure, coordination with the anesthesia team was crucial. During the intubation or the extubation, only personnel considered essential to the procedure remained in the surgical room, while all non-essential staff leaved the room and returned after the airway was secured.

We also payed particular attention and used adequate protection when transferring patients. Clear protocols were established with the anesthesia department, nursing staff, recovery unit personnel and infection control team .Adequate protection when transferring patients is critical. Non-intubated patients were transferred while wearing a surgical mask. The administration of oxygen was performed by face mask over the surgical mask. Intubated patients were transported with an ICU ventilator (dry circuit, filter in place) in order not to interrupt the closed circuit. Every healthcare worker who participated in the patient transfer wore PPE. Also nurses performing post-operative activities received appropriate PPE.

Particular attention was paid to tracheotomized patients. In these cases, a closed suction system was used to minimize secretions during nursing procedures. At this specific time, managing cancer patients has become an even more difficult challenge as cancer patients represent a high-risk group in the COVID-19 pandemic emergency. These patients are already highly vulnerable to infection due to their underlying tumor disease and generally immunosuppressed status, which implies an increased risk of serious viral complications, including ICU admission or even death.

In fact, in some Italian and European cities, the media reported numerous reductions in medical or surgical treatment for cancer to prioritize seriously ill COVID-19 patients [22]. During the COVID-19 pandemic, head and neck cancers present a particularly complicated challenge for the head and neck surgeon. Literature studies widely report that these head and neck cancers (laryngeal tumors, advanced nasopharyngeal tumors, base tongue and more generally oral cavity tumors) can produce severe breathing difficulties if not treated [23]. This problem is particularly relevant for patients with advanced disease (stage III or IV) laryngeal cancer, who represent 60% of the cases that come to our attention. Given the unique physiological function of the larynx, there are a number of common symptoms that may favor further intervention for laryngeal cancer, such as hoarseness, dysphonia, swallowing and dyspnea [24]. In fact, dyspnea is present in all the most severe cases of COVID-19 patients. For this reason, early diagnosis and urgent surgical treatment are mandatory not only to ensure the highest 5-year survival rate, but also because a COVID-19 infection in such patients could be fatal in a very short time.

At the INT-Pascale, during the COVID-19 epidemic (21 February-21 May 2020) there was no reduction in the treatment of head and neck cancers, with particular attention to cancers laryngeal (open surgery, endoscopic LASER) and advanced tumors of the base tongue and oral cavity. Transoral robotic surgery (TORS) was not suspended, also because a review of the literature showed that in selected cases TORS could reduce hospitalization times. All respiratory protection procedures have been adopted and particular attention has been paid to cleaning the robot and console [25].

One hundred twenty-nine (129) major surgical procedures were performed for head and neck malignancies; these included 7 total laryngectomies and bilateral neck dissection, 22 partial laser laryngectomies, 6 emergency tracheostomies, 38 major surgeries, 14 TORS procedures. Only surgical procedures in which the aerodigestive tract was not involved (i.e. parotidectomy), were not subject to the operating room COVID protocol.

Furthermore, the head and neck surgeon played another crucial role in this pandemic emergency phase.

ICU patients undergoing prolonged intubation often require safer management of the airways, so in these cases it was necessary to perform a surgical tracheotomy which, if performed in the first 7 days after orotracheal intubation, is associated with a reduction of mechanical ventilation and hospitalization in ICU, as well as with the mortality rate [26]. All of these procedures required a very high level of containment and were performed under general anesthesia rather than in a sedated patient, as there is a large production of droplets from the stoma during direct access to the trachea [27].

Finally, H&N physicians contributed to the identification of paucymptomatic patients whose symptoms were mainly anosmia and ageusia [28].

### Abdominal surgical oncology during COVID-19 pandemic

Surgical oncology at the INT-Pascale has not been stopped by the COVID-19 pandemic but prioritization has been necessarily taken into account to face the need for reduced resources and modified admission and management strategies. Being a potential COVID-free hospital, in accordance with a shared strategy, all the Abdominal Surgical Oncology Units tried to identify uniform approaches to minimize the impact on quality and timing of surgery for patients enlisted for elective treatment [29]. Screening of patients for COVID-19 has been widely adopted to identify suspected or positive cases in order to postpone surgery and maintain the safety of both patients and professionals.

Telemedicine, smart-working and web-based multidisciplinary meeting have been promptly adopted to guarantee social distancing. Surgical wards have been remodulated reducing the number of beds for rooms, relatives’ visiting hours have been abolished and daily telephone call with contact persons for each patient have been guaranteed. Rotation and workload have been limited having no more than two doctors working in the ward. Nurses have also entered a rotation system to reduce the risk of infections.

### Surgical approach

Minimally invasive surgery, both robotic and laparoscopic, was conducted with a high safety profile through the use of new insufflation systems, such as AirSeal® iFS (CONMED, USA): it offers continuous smoke evacuations through Ultra Low Penetrating Air (ULPA) Filter that allows the capture of particles > 0.01 μm (the CoV-2 virus has a size of 0.06–0.14) [30, 31]. Moreover, this new type of 3-way insufflators keep the abdominal pressure low and has an integrated active smoke evacuation mode that prevents the risk of transmitting SARS-CoV-2 from aerosols. Extensive use of appropriate PPE was ensured for both surgeons and nurses [32].

### Timing and delay of surgery

In general, any delay in the surgical approach in patients with abdominal cancer could have an impact on survival, but a prioritization program should consider not delaying patients who

1. have completed neoadjuvant treatments,
2. have highly biological aggressive tumors, and
3. do not have alternative strategies for their tumors.

### Specific sites management

#### Colorectal cancer

Prioritization of cases has followed fully shared decisions by the multidisciplinary team taking into account the experience of other centers in Italy [33]. Patients with nearly obstructing or bleeding colonic cancers have received upfront surgery within the expected 30 days from the diagnosis; a similar approach has been guaranteed for patients with complicated high and low rectal cancers. Stoma formation has been selectively adopted to reduce postoperative complications in high risk patients. Patients with rectal tumors with little or no response to neoadjuvant treatment have also been operated on. Surgical approach for locally advanced rectal cancer with major or complete response after neoadjuvant treatment has been delayed.

Polyps, asymptomatic carcinoids, and prophylactic surgery for inherited diseases have been postponed. Continuation of neoadjuvant chemotherapy was planned for those patients with locally advanced colon and rectal cancers according to dedicated protocols. Delaying the progression of the disease pending surgery. Short-term radiotherapy has also been considered for early-stage distal rectal cancer, mainly in elderly patients.

The endoscopic stent for obstructive tumors has also been adopted as a “bridge for surgery” in patients at high anesthetic risks.

#### Esophageal cancer

For all histological types, patients with stage 1 disease have been postponed: stages II and III have been proposed for neoadjuvant therapy. Patients with stage IV disease have been placed on integrated treatment pathways or have been relieved for symptoms and complications.

#### Gastric cancer

Only early cancers have been postponed. Patients with locally advanced diseases have been discussed for the neoadjuvant approach which is a standard for many clinical situations. Neoadjuvant chemotherapy has been continued in those with minimal toxicity to delay surgery.

#### Pancreatic cancer

Neoadjuvant chemotherapy has been identified as the best approach for all uncomplicated cases. The surgical approach has been guaranteed to patients already treated with chemo-chemoradiotherapy. Symptomatic patients have been offered endoscopic stents and gastro-jejunal bypass.

#### Liver tumors

The surgical approach, with both minor and major resections, has been guaranteed in patients with liver metastases already treated with chemotherapy in a neoadjuvant approach. Careful selection of patients made it possible to postpone those at high risk of postoperative complications. Local ablation for primary and metastatic tumors has been performed regularly.

#### Retroperitoneal sarcomas

The surgical approach, even in the case of extensive multivisceral resections, has been guaranteed in patients with localized and resectable tumors with minimal delay. Biologically aggressive tumors have been incorporated into integrated treatment pathways..

#### Peritoneal neoplasms

Because peritonectomy followed by hyperthermic peritoneal chemotherapy is a very aggressive procedure, patients with primary and metastatic peritoneal tumors were postponed.

### COVID-19 and radiotherapy

Radiotherapy is a life-saving treatment and must be guaranteed for cancer patients for whom it is indicated, as requested by several oncological Scientific Societies [34]. It is estimated that 60% of cancer patients should include radiation therapy as part of the treatment.

Radiation therapy plays an important role in cancer patients as neoadjuvant treatment before surgery, adjuvant therapy after a primary treatment of surgery, concomitant to other therapies for synergistic enhancement of systemic treatments and exclusive treatment. In these cases, failure to administer radiotherapy adversely affects local control and overall patients’ survival. Finally, in patients with bone or brain metastases, bone marrow compression and mediastinal disorders palliative radiotherapy significantly improves pain control and quality of life.

During COVID-19 outbreak radiation therapy departments could represent a site of exposure for patients and operators because radiotherapy usually involves daily treatment for days or weeks in a closed environment (many radiotherapy centers are situated in basements). In this respect, one of the main critical problems was the management of patients’ flow and their caregivers who access radiotherapy facilities. For this reason, it was necessary to develop specific protocols and protective measures to prevent the spread of COVID-19 during radiation procedures and reduce the overcrowding of facilities and the risk of infection among fragile and vulnerable patients and staff [35, 36].

All the clinical activity, comprising CT-simulation, were re-scheduled in order to reduce the risk of contagion for both patients and staff. During emergency phase 1, doctors, physicists and administrative staff work in two shifts with rotation assignments and all activities that did not require on-site presence (such as phone calls and physicists planning) were carried out via telemedicine. In particular, it was scheduled the presence on-site of one medical physicist supported by colleagues in remote working. Live staff meetings were no longer performed and staff workstations were located in different areas to allow social distancing. Adequate interval time between CT-simulation and start of the radiation therapy was guaranteed with no further delay or extended radiotherapy interruption. As results of our strategies, there was no reduction in terms of number of patients treated during COVID-19 outbreak, but there was a reduction in the treatment waiting list. In the first trimester of 2020, 614 treatments were performed in comparison to the 598 treatments performed in the first trimester of 2019.

**Fig. 3:**
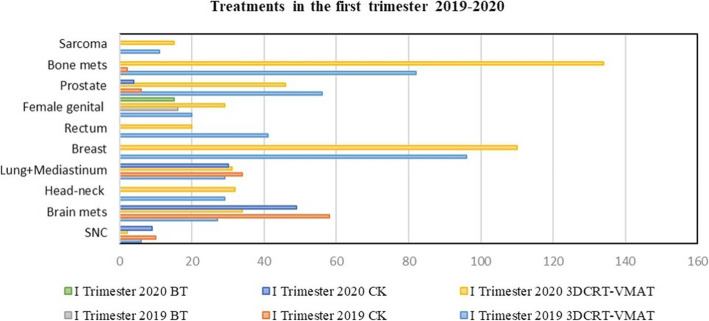
Number of patients treated with Brachytherapy (BT), CyberKnife (CK), Three-dimensional Conformal Radiation Therapy (3DCRT) and Volumetric Modulated Arc Therapy (VMAT) in the first Trimester of 2019 and 2020

Figure 3 shows the number of treatments delivered in the first trimester of the 2 years (2019 and 2020) stratified by pathology. Hypofractionated regimens have been favored [37, 38]. Moreover, collaboration with Ascalesi Hospital in Naples, have favored the treatment of a greater number of patients with an increased number of breast cancer and bone metastasis treated in 2020 compared to 2019.

In general, treatments that included neoadjuvant hormonal therapy were postponed, whereas radiation therapy was ensured for patients with in situ cancers and for adjuvant or concomitant radiation therapy candidates.

**Fig. 4:**
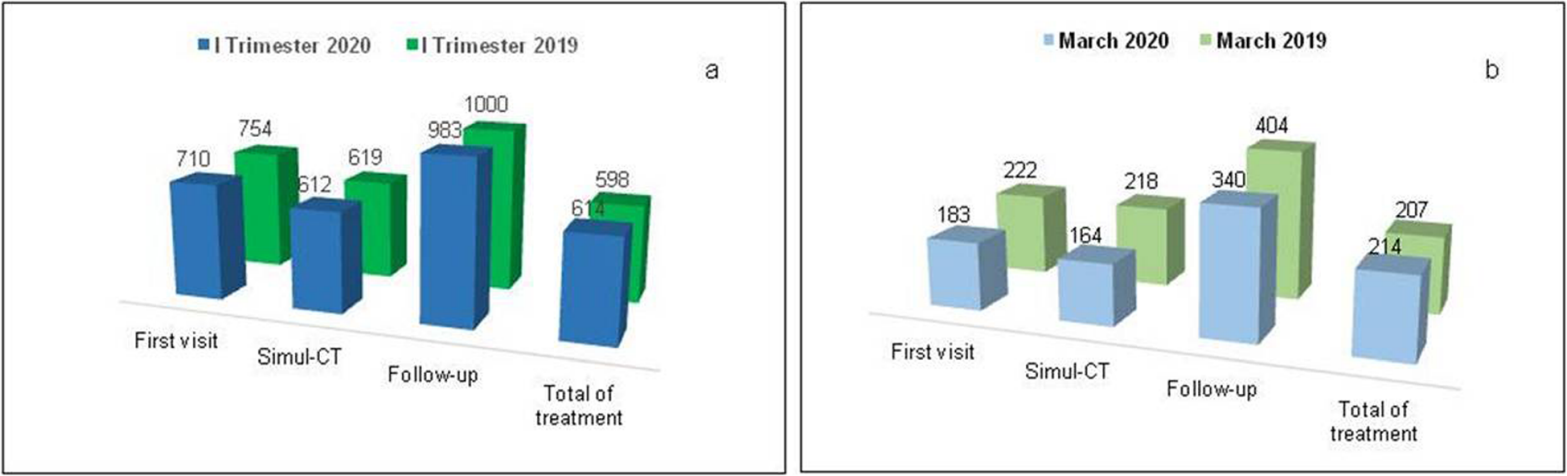
Activities performed in Radiotherapy Department, number of first evaluation, simul-CT and follow-up in the first Trimester (**a**) and in March (**b**) of 2019 and 2020

Figure 4 (a,b) compares the activities carried out in the first 3 months of 2019 and 2020 and in March 2019 and 2020, respectively. As observed in Fig. 4a, there was a slight reduction of first clinical evaluation in the first trimester of 2020 compared to 2019. This was due to the postponement of radiotherapy access for patients eligible to neoadjuvant systemic treatment. Instead, thanks to collaboration with Ascalesi Hospital, every patient who was a candidate for radiation treatment that could not be postponed was treated. Figure 4b shows a reduction of activities in March 2020 compared to 2019, as consequence of Italian lockdown for COVID-19 outbreak emergency measures introduced by the government on March 9. This reduction did not impact patient treatments and did not significantly change the quarterly data, thanks to a notable increase in activity in January and February. Following a multidisciplinary evaluation, radiation treatment was delayed for several months only in patients for whom neoadjuvant hormone therapy was possible. The reduction in radiotherapy activity affected the number of first visit assessments, the number of CT simulations as only patients who had to quickly begin radiotherapy were simulate, and the number of follow-ups.

**Fig. 5:**
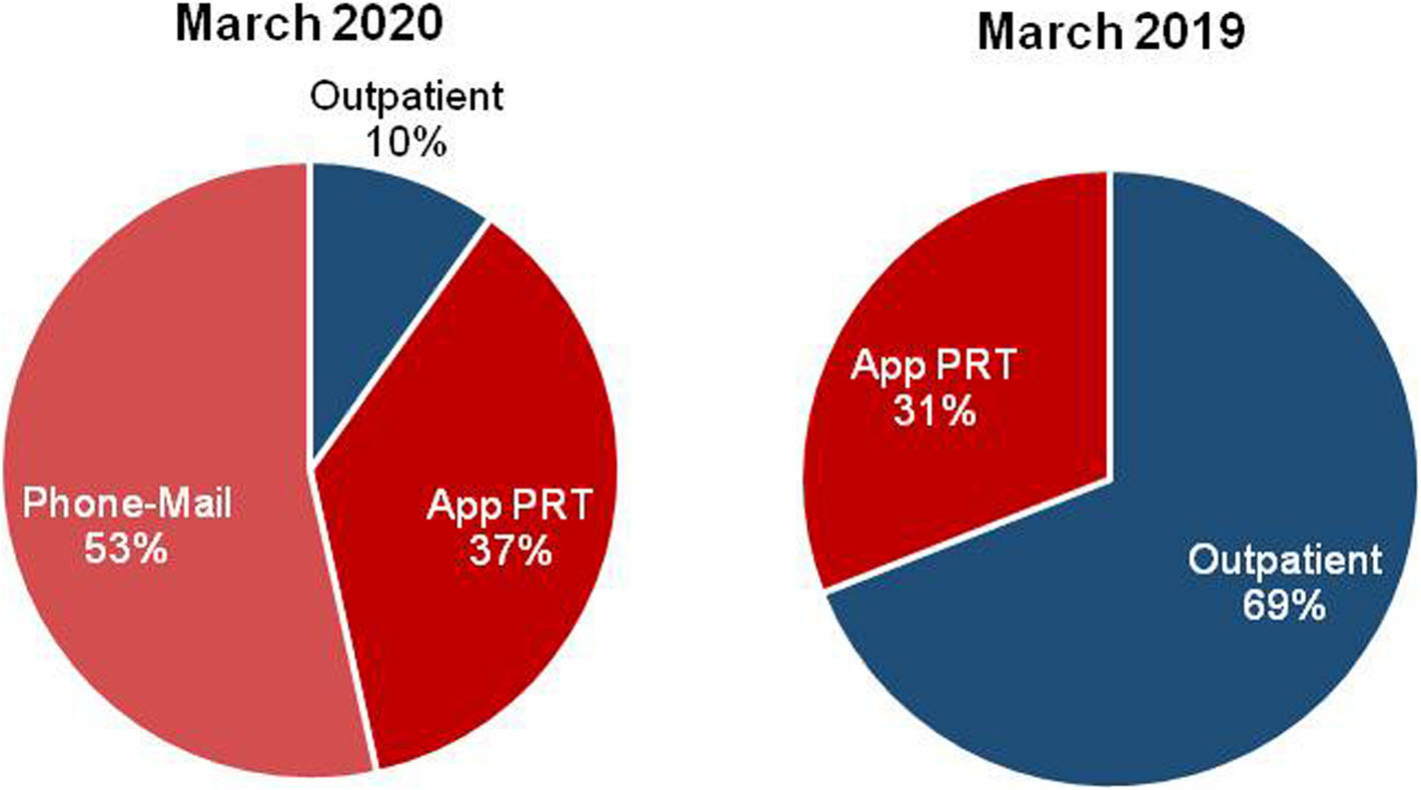
Types of follow-up performed in March 2020 (**a**) compared to March 2019 (**b**). Experience of outpatient follow-up and telemedicine (App and phone/mail)

With regard to follow-up visits, further clarification is required respect to graphic shown in Fig. 4b. Radiation oncologists, using telemedicine (telephone calls, app and emails), carried out 340 follow-up visits. Thirty-three outpatient visits were performed in the first week of March 2020, then, after government lockdown, each patient was telematically guided to share diagnostic tests with the radiation oncologists. Many prostate patients’ follow-up were performed via our Prostate RadioTherapy App, which has been used as routine clinical practice for several years. The app collects and stores biochemical parameters, genitourinary and gastrointestinal toxicity, sexual activity and quality of life questionnaire data entered into the database. Thanks to the app, the radiotherapy staff constantly follows more than 100 patients with prostate cancer and, during the COVID-19 emergency, has proven to be an important tool for the management of these patients. Therefore, in March 2020 of the total 340 follow-ups only 33 access (10%) were on-site (Fig. 5a), while in March 2019 of the total 404 radiotherapy follow-ups 279 (69%) were carried out in the Department (Fig. 5b). These data show that telemedicine was applied in 90% of cases with a consequent drastic reduction in patients’ access to the facility.

Of note, some patients were unable to perform clinical exams because of COVID-19 emergency. As consequence, it was recorded a reduction of about 16% of total follow-up visits in March 2020 compared to the March 2019. Our data show the effectiveness of these approaches, suggesting that health services could be carried out in telemedicine mode also outside of the epidemics, resulting in savings on sky rocking health costs.

### Diagnostic procedures

- Diagnostic imaging

The INT-Pascale Diagnostic Imaging units has contributed to identify COVID-19 patients and to evaluate the lung parenchyma involvement. Although our Institute is not a COVID-19 hospital (not dedicated to hospitalization of SARS-CoV-2 infected patients) all diagnostic units have been involved in identifying potential COVID-19 patients for their isolation and subsequent transfer to COVID-19 hospitals. All hospitalized patients with suspicion of COVID-19 for symptoms of acute respiratory infection, while waiting for the swab collection and PCR analysis, have been assisted in hospital ward and subjected to chest X-ray at patient’s bed to evaluate the involvement of the lung parenchyma. Chest X-ray examination, although not offering highly specific findings, provides a first overview of the patient and may direct differential diagnosis to other possible causes of pulmonary parenchymal involvement other than COVID-19 infection [39]. Bandirali et al. at the Codogno Hospital, where the Italian COVID-19 epidemic was recognized, were able to identify chest x-rays abnormalities highly suspicious for COVID-19 pneumonia in 100 of 170 (59%) patients [39]. Involvement was bilateral in all cases: in 54% of patients the involvement was symmetrical. Furthermore, chest X-ray at the patient’s bed is a valid tool for the evolutionary monitoring of pneumonia in hospitalized patients and in intensive care [40, 41]. Following X-ray, COVID-19 suspicious cases have been subjected to the nucleic acid amplification test of the respiratory tract or blood specimens using reverse transcription real-time polymerase chain reaction test (RT-PCR). In case of positive RT-PCR, patients have been transferred to regional infectious disease centers dedicated to COVID-19 management. Since the X-ray suspicion of COVID-19 infection, patients have been isolated in sub-intensive rooms and monitored with the appropriate diagnostic and laboratory test, waiting for the PCR results and eventual transfer to COVID-19 hospitals.

In this context, recent results have revealed the efficiency of some imaging methods in the management of COVID-19 disease. The chest ultrasound (POCUS - Point-Of-Care UltraSound) can be performed by the intensivists at the patient’s bed and represent a valid monitoring tool to evaluate the effectiveness of the prono-supination maneuvers [41–43]. POCUS can reduce the use of diagnostic imaging resources, risk of contagion for personnel, and sanification time. Moreover, it helps in POC decision for critically ill patients. On the other hand, the ultrasound scan itself requires prolonged contact between the operator and the patient, and has other contraindications, including dependence on operators [41–43]. Computed tomography (CT) examination has been used extensively in China and now worldwide to evaluate the grade and the extension of the viral pneumonia by COVID-19 especially in the follow-up [44–46], also supported by Artificial Intelligence algorithms [47, 48]. Bilateral distribution of ground-glass opacities, with or without consolidation, in posterior and peripheral lungs was initially described as a characteristic feature of COVID-19 [47, 48]. However, several radiological organizations do not recommend CT as primary screening tool for COVID-19 [49–53]. Moreover, safely using CT to study COVID-19 patients is logistically challenging and can overwhelm available resources. Even with proper cleaning protocols, healthcare professionals and CT scanners could become vectors of infection to other vulnerable patients who require CT imaging.

- Diagnostic procedures (Laboratory Medicine)

The Laboratory Medicine Unite of the INT-Pascale has been involved since the beginning of the epidemic in the identification of the COVID-19 patients as well as on the early SARS-CoV-2 exposed health workers. The promptly organized COVID-19 crisis unit of the INT-Pascale, under the Director General (Dr Attilio Bianchi) solicitation, established a program of health surveillance for all patients as well as healthcare workers with travel, exposure or symptoms history suggestive for infection with SARS-CoV-2. Moreover, molecular methods were selected as a gold standard and immunology tests (i.e. serology and rapid antigen tests) recognized as supplementary diagnostic tools [54]. The Laboratory Medicine personnel (medical director), first of all, identified and implemented the biosafety conditions recommended by World Health Organization’s interim guidance [55]. A validated internal protocol including pre-analytical, analytical and post-analytical phases (from sample transportation to elaboration of medical reports) was derived from it.

Molecular testing, as recommended by the World Health Organization, has been used as reference method for the identification of SARS-CoV-2 infectious cases [56]. Nucleic acids extraction and subsequent Real-time PCR detection of SARS-CoV-2 RNA from nasopharyngeal swabs were performed [57]. The Charité algorithm (Berlin, Germany) worked out by Christian Drosten and colleagues, based on Real-time PCR detection of E and RdRp genes was used as a reference method [58]. Molecular testing found application primarily in early stages of disease and in detecting asymptomatic carriers.

Rapid immunochromatographic assays, used as an additional diagnostic procedure, have shown the advantage of rapid results times and low cost detection. However, they were likely to suffer from poor sensitivity and limited specificity making them more useful in monitoring positive subjects than in the initial diagnosis.

According to good microbiological practice and procedure, initial processing (before virus inactivation) of specimens from cases with suspected or confirmed COVID-19 infection has taken place in a validated biological safety cabinet. Either propagative or non-propagative diagnostic laboratory work has been conducted in a laboratory area following biosafety standards. Only staff trained in the relevant technical and safety procedures has been admitted in handling and processing hazardous specimens, according to internal protocol. In particular, two laboratory technicians were provided with appropriated personal protective equipment and assigned to these activities. Management of assays interpretation, results and medical reports was committed to a single healthcare worker, in order to standardize the post-analytical phase. Patients’ results were made available promptly to wards by means of “order entry” data visualization, whilst reports relative to hospital personnel were transmitted only to the Medical Director of the surveillance office.

Diagnostic workflow applied in our Laboratory provided three steps:

- rapid immunochromatographic assays to detect SARS-CoV-2 IgM and IgG from plasma samples of patients and healthcare workers from 31.03.2020 to 15.05.2020;
- automated qualitative electrochemiluminescence immunoassays (ECLIAs) to detect SARS-CoV-2 antibodies from serum samples of healthcare workers from 11.06.2020 to date.
- Real-time PCR detection of SARS-CoV-2 RNA from nasopharyngeal swabs of patients and healthcare workers from 20.05.2020 to date.

At the beginning of the surveillance, rapid immunochromatographic tests allowed to screen 1991 patients (96 of which resulted positive) and 1050 healthcare workers (48 of which positive).

Subsequently, healthcare workers underwent further ECLIAs screening, revealing 26 positive subjects out of 1547 tested.

**Fig. 6:**
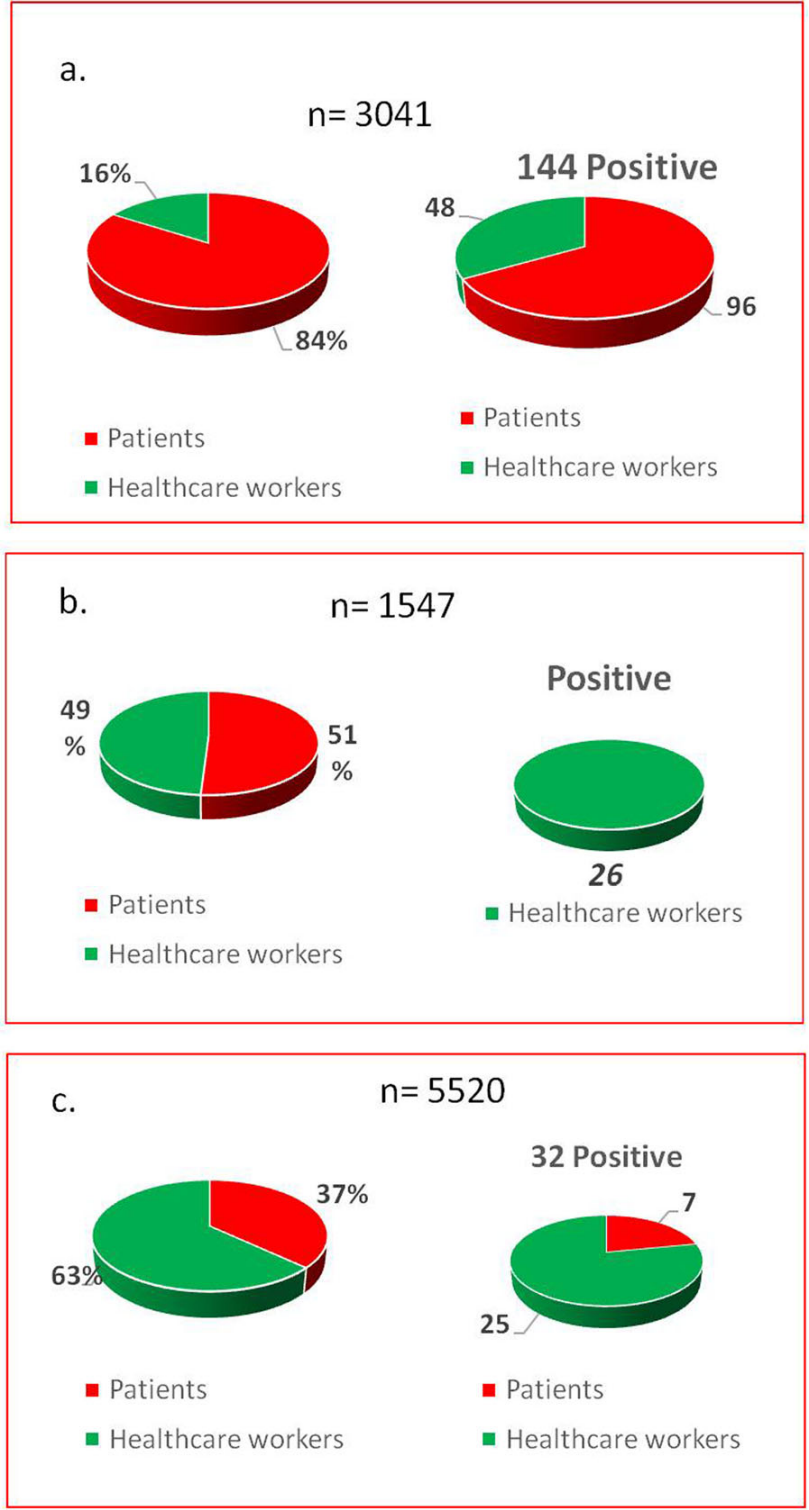
Laboratory Medicine data for Patients and Healthcare workers by (**a**) rapid immunochromatographic assay; (**b**) electrochemiluminescence immunoassays (ECLIAs); and Real-time PCR on nasopharingeal swabs

Early detection and quarantine of positive cases allowed to find very low percentages of positive SARS-CoV-2 subjects to molecular tests from nasopharyngeal swabs: 25 positive from healthcare workers and 7 positive from patients out of 5520 nasopharyngeal swabs analyzed (Fig. 6).

All the efforts of the staff involved in COVID-19 diagnostic procedure have been directed to guarantee an adequate turnaround time, in order to furnish results in a shorter time as possible. As a matter of fact, the element “time” has been crucial to subject promptly to home quarantine personnel identified as positive or move to reference hospitals affected patients.

- Diagnostic procedures (Pathology Unit)

The Pathology Unit, involved in all extemporaneous exams on fresh samples diagnostic procedures, in particular tailoring radicality in demolitive surgery, has promptly chosen and implemented special and extraordinary precautions for management and handling of biological samples. The SARS-Cov2 virus has been identified mostly in tissues and biomaterials of lung origin, but, although more rarely, in other biomaterials, including blood [59]. Therefore, in the Pathology Unit, given that the SARS-CoV-2 can be present in all surgical and cytological biomaterials, fresh or inadequately fixed, all biological samples have always been considered potentially infected and consequently all operators have been equipped with adequate personal safety devices (PSD).

The handling of biological samples regards in particular the “acceptance” and the “processing” steps of tissue and cytological samples. For the acceptance it must be distinguished whether the samples are coming from the hospital operating theatre or from second opinion outpatients: i) for surgical and cytological samples, in particular pulmonary samples, access to the pathology laboratories of surgical operators, equipped with the PSDs, and the delivery of the samples to selected, dedicated technician was provided at set times. ii) for the second opinions, a Triage structure was set up in front of the Pathology Unit laboratory, to monitor the temperature of the patients who, also equipped with a mask and at a safe distance, arrive with already prepared biological samples. All biomaterials that arrived in the laboratories were always accompanied by a request containing the clinical information relating to the SARS-Cov-2 infection.

For the “Processing” of biological samples they have been all considered at microbiological risk. Samples have been fixed immediately in 10% buffered formalin for at least 24 h at a temperature between 25 °C and 37 °C, following the recommendations of the SIAPEC-IACP (*Società Italiana di Anatomia Patologica e Citologia Diagnostica-Divisione Italiana della International Academy of Pathology*) [60]. This procedure, preferring the use of formalin, which favors the containment of biological risk compared to the chemical one, allows to inactivate almost all the viral particles in the tissues. Subsequent handling of the samples was carried out under hoods with biosecurity characteristics to contain the biological risk.

Intraoperative histological procedures, which require handling of fresh tissue specimens, particularly lung lesions, are not recommended for patients who are positive or suspected for SARS-CoV-2 [60]. In other cases, the samples have been immediately processed with the appropriate precautions and the expected PSDs. However, diagnostic imaging strategies were implemented during the pre-operative phase, to minimize the need for intraoperative examinations.

## Guidelines/recommendation for selected cancers

### Guidelines/recommendation for breast cancer

Breast cancer is a widespread disease of varying severity and stage, affecting women of all ages with a relevant emotional component. In order to ensure breast cancer continued care and treatment and protect patients and healthcare professionals from the COVID-19 pandemic, a priority program has been developed to minimize patient hospitalization and ensure optimal patient care. In this perspective, instead of the proposed general deferral strategy [17], it was decided not to defer breast cancer treatment that have been classified as at medium/high priority by ESMO (https://www.esmo.org/guidelines/cancer-patient-management-during-the-COVID-19-pandemic/breast-cancer-in-the-COVID-19-era), while avoiding or delaying low priority interventions only. Patients and staff safety procedures have been referred to the general protection policy of the hospital.

In particular, specific programs were adopted for early and for metastatic breast cancer.

- Early Breast Cancer (EBC)
  - Surgical treatment for confirmed or highly suspicious (BIRADS ≥4) invasive EBC has been delivered as planned, while benign/DCIS, delayed reconstructions or prophylactic mastectomy have been deferred. Upon specific patient request, low risk, Luminal-like tumors have been put on six-months endocrine neo-adjuvant treatment with surgery postponing.
  - Adjuvant and Neo-adjuvant treatment have been usually delivered as planned. For HER2-positive tumors, patients have been offered the option to continue Trastuzumab treatment at their home upon completion of the planned chemotherapy. This was possible thanks to an already implemented project of home delivering of subcutaneous Trastuzumab (HerHome). Lower risk patients (particularly if elderly and/or with cardiovascular comorbidities) who feared for the COVID-19 were also given the option to stop Trastuzumab after completion of the first 6 months of treatment. This was based on a methanalysis of clinical trials showing that shorter than standard 1-year treatment was associated with a marginal and non-statistically significant reduction of disease-free survival [61].
  - Patients on chemotherapy received prophylactic treatment with G-CSF to minimize the risk of neutropenic infections.
  - LHRH-analogue administration, when indicated, has been prescribed as regular monthly injections, as it is usually self-administered at home by our patients.
  - Follow-up visits of disease-free patients have been postponed/cancelled by implementing remote verification of health status (including phone calls, emails and communication apps).
- Metastatic Breast Cancer (MBC) treatment
  MBC is a very heterogeneous disease and, thus, optimizing treatments in the COVID19 era required a careful case-by-case evaluation of the trade-off between potential benefits and harms of the different options.
  1. First-to-third line standard treatments have been delivered as planned, since they have worthwhile impact on either OS, PFS or QoL, particularly for HER2-positive and for Luminal-like tumors.
  2. Treatment of visceral crisis has always been regarded as a high priority, since it aims at avoiding an impending risk of dying.
  3. Whenever possible, oral treatments have been preferred if this did not negatively impact on prognosis.
  4. Whenever possible, chemotherapy schedules have been modified to reduce the frequency of hospital access.
  5. Hospital visits have been reduced by implementing remote management of symptoms and adverse events (including phone calls, emails, communication apps). Also, disease restaging has been delayed as much as possible in asymptomatic patients and/or with lower disease burden.
  6. The combination of everolimus and exemestane in luminal-like MBC has been deferred or avoided because of the immunosuppressive effect of the m-TOR inhibitor.

### Recommendation for urological cancers

Appropriate surgical timing is generally relevant for cancer disease. Delay in surgical treatment of non-emergency cases and in the setting of urological surgeries might increase the risk of sub-optimal oncologic outcomes in cancer patients or the risk of infection and urosepsis in other cases. Surgical procedures, however, can directly expose health workers, including surgeons, to the SARS-CoV-2 from non-diagnosed, paucisymptomatic or asymptomatic patients during laparoscopic and robotic procedures. In order to timely and safely treat patients and to protect robotic surgery healthcare workers, the following preventive measures, based on the most recent scientific evidences, have been defined and implemented at the INT-Pascale when performing robotic procedures on patients potentially or proven COVID-19 positive.

1. General protection

All employees and patients referred to the hospital for any kind of urological cancers received a general health screening, regardless if they were symptomatic or not, at the check point located at each hospital entrance. To prevent infections of health workers, all medical personnel had to follow triage procedures. General health and COVID-19 screening have been performed to all candidate patients to undergo minimally invasive surgery. In case of COVID-19 positive patient, surgical procedures have been postponed except for urgent and emergency procedures. The latter have been performed in dedicated operating room following the INT-Pascale recommendation. In case of negative COVID-19 result, considering the possibility of false negative, all necessary protection tools and general recommendations to reduce COVID-19 transmission have been implemented [62].

2. Patient selection

In order to ensure an adequate number of medical personnel involved in the COVID-19 emergency, including internists, anesthesiologists, or nurses, all elective surgery that could have been delayed without any risk for the patient has been postponed. Moreover, this measure was also aimed at limiting the consumption of medical equipment, useful to deal with the COVID − 19 emergency, such as masks, sanitizing gel or ICU for SARS-CoV-2 positive patients, as well as to ensure the ordinary course of emergency cases requiring the use of operating rooms and intensive care units [63].

3. Prevention and management of aerosol dispersion

In case of surgery that could not be postponed, the possibility of surgical smoke formation and the spread of small viral particles during laparoscopic procedures had to be kept in mind [64, 65]. As a consequence, any laparoscopic or robotic surgery was performed only when needed and at the lowest intra-abdominal allowed pressure. In this regard, the use of intelligent integrated flow systems was really useful.

4. Operation technique

Attention was paid to ultrasonic scalpels or electrical equipment, commonly used in minimally invasive surgery, able to produce large amounts of surgical smoke, considering that low-temperature aerosol generated from ultrasonic scalpels or scissors cannot effectively inactivate the viral particle present in the first.

5. Pneumoperitoneum disinflation

It became mandatory to confirm and verify the complete and correct disinflation of the pneumoperitoneum at the end of the procedure. In fact, due to the poor mobility of the gas in the pneumoperitoneum, the aerosol formed during the operation tends to concentrate in the abdominal cavity [66]. The sudden release of trocar valves, the non-hermetic exchange of instruments or even small abdominal extraction incisions could potentially expose the surgical team to pneumoperitoneum aerosol. This evidence further supports the use of systems with integrated active smoke evacuation mode (https://www.sages.org/recommendations-surgical-response-COVID-19/). In contrast, classic insufflation systems that are not provided with the active smoke evacuation mode or other filters can expose the operators to a greater risk of SARS-CoV-2 aerosol transmission.

6. Urinary transmission

Persistence of SARS-CoV-2 nucleic acid in urine has been reported [67]. Although this data does not clearly justify a correlation between urine spillage and virus transmission in the aerosol during robotic procedures, and no evidence of disease transmission through the urine has been shown, urethral or ureteral catheterization during the laparoscopic and robotic procedures has been executed with caution, particularly if pneumoperitoneum was already induced.

7. Operating staff protection

The whole surgical team (including surgeons, anesthetists and nurses) have routinely adopted adequate protection devices. Goggles, FFP2 mask and disposable body suit were used also in case of any minimally invasive procedures performed during the COVID-19 pandemic. In order to avoid any contact with the droplets, sealed visor masks were worn by the console surgeon and the console head support between the cases was thoroughly cleaned.

### Guidelines/recommendation for pancreatic cancer

A consistent delay in effective surgical treatment of solid tumor has already been demonstrated to negatively affect tumor stage and oncological outcomes for several cancers type, and in particular for hepatobiliary malignancies [68]. The doubling time of pancreatic ductal adenocarcinoma (PDAC) is known to be remarkably rapid, as well as its aggressive biological behavior and rapid potential for metastatic spread [69]. Therefore, rapid access to potential radical resection is of fundamental importance for this pathology. However, despite the recent optimization of peri-operative optimization protocols, surgical techniques and anesthesiological treatments, morbidity and mortality after pancreatic resections are still not negligible especially in the case of cephalic localization [70]. In addition, patients with surgical resection are likely to require postoperative treatment in intensive care units, mainly reserved for COVID-19 positive patients during the pandemic. Therefore, the benefit of a prompt surgical resection has to be counterbalanced to the higher risk of viral exposition and development of severe complications in immunosuppressed cancer patients, frequently affected also by cancer-related malnutrition [71]. For PDAC and periampullary tumors, the INT-Pascale policy has been to prioritize symptom reliefs in patients with blood transfusion and biliary, endoscopic or percutaneous decompression, as most appropriate. An effort was made to obtain a histological diagnosis in patients without a defined diagnosis. Therapeutic choice was then achieved through online digital multidisciplinary consultation meetings favoring neoadjuvant therapy strategies in locally advanced and borderline resectable cases. Surgical resection was decided taking into account for each patient tumor location, nutritional status and comorbidities. Prehabilitation strategies were implemented and normalization of nutritional status prioritized. PDAC located to the body and tail of the pancreas were scheduled for surgery in case of low operative risk and low comorbidities burden. Interim chemotherapy was an option in case of patients at a high risk of developing postoperative complications. Patients with cephalic lesions deserving a pancreatoduodenectomy were scheduled for surgery only in case of tumors smaller than 2 cm [72]. This subjects (for low comorbidities burden, optimal nutritional status and younger age) are the more likely to benefit from a radical resection and are at lower risk of developing postoperative complications, taking in consideration the availability of beds in the intensive care units for the COVID-19 pandemic. For larger cephalic lesions interim chemotherapy and neoadjuvant strategies were preferred. In case of Gastroenteropancreatic NeuroEndocrine Tumors (GEP-NET) interim chemotherapy or somatostatin analogues administration were preferred due to the lower biological aggressiveness in comparison to PDAC.

### Guidelines/recommendation for liver cancer

The Hepato-biliary Units continued to provide high quality assistance for oncological patients during COVID-19 pandemic, with some variation on the clinical care path, after online digital multidisciplinary consultation meetings.

Surgical approach, both with minor or major resections has been guaranteed in patients with liver metastases already treated with chemotherapy in a neoadjuvant approach. Careful selection of patients has allowed to postpone those at high risk of postoperative complications. Patients undergoing Hepato-biliary surgery had a strictly correlation with available post-operative intensive care beds. The consequent reduction of post-operative intensive care beds influenced the surgical planning.

This multidisciplinary decision was made to give the chance to better planning the use of post-operative intensive care beds and, at the same time, ensure the best possible quality of oncological care. Indeed, evolution of chemotherapy for stage IV of colorectal disease, has showed important clinical results [73–76].

In hepatocellular carcinoma (HCC), chemotherapy is not a viable alternative to the surgical approach and, due to the underlying cirrhosis, this type of patient is at high risk of postoperative complications [77, 78]. For all this reason each single patient was strictly evaluate by multidisciplinary team to guarantee the most correct surgical indications, using more frequently as an upfront treatment locoregional procedure like embolization, chemoembolization, radioembolization, or percutaneous ablation, selecting for surgery only patients in very good general clinical conditions.

For their aggressive nature, Cholangiocarcinoma and gallbladder cancer, requested unchanged approach during pandemic: timely surgical intervention with no delay to obtain a good oncological results.

### Interventional radiology in oncology during COVID 19 pandemia

Interventional radiology (IR) is an essential service and provides advanced image-guided treatments of cancer patients also to critically ill ICU patients. In the first quarter of 2020, despite the reduction of total activity, 1.049 procedures, including diagnostic biopsies, have been performed with an increase in emergency activity for epato-gatro-intestinal bleeding, and sub-emergency IR procedures such as thoracic, biliary and abdominal drainage for abscess, unsealed fistula of the anastomosis or ascites.

The main goal has been always to provide the highest quality services at the best safety conditions to protect patients and worker team. For such reason all patients, although prevalently uninfected COVID-19 patients, have been routinely instructed about mask- wearing and hand hygiene, with the COVID-19 prevention warning.

Although, the angio-suite rooms at the INT-Pascale are provided of a negative-pressure ventilation with high-efficiency particulate-absorbing (HEPA) filtration, which should represent an efficient barrier for virus transmission, further aspects need to be taken into consideration.

Safety measures for patients:

1. Triage of all inpatients and outpatients.
2. Physical and temporal separation of inpatient and outpatient cases, which have been performed in different two separate angio-suite rooms at different times (alternate days, different hours).
3. All patients had to wear surgical mask and disposable gown.

Safety measures for health workers:

1. For all staff wearing a surgical mask has been mandatory in clinical areas (i.e. procedure rooms, clinics, patient waiting rooms, and other areas) for the direct interaction with patients who could be shedding virus also when minimally symptomatic and even asymptomatic.
2. Enhanced personal protection equipment (PPE) has been calibrated to the risk level. All staff have been fit-tested for N95 masks.
3. During aerosol-generating procedures, full PPE (cap, goggles or face shield for eye protection, N95 mask, full gown, and gloves) has been required for Health Care Workers (HCW) regardless of patients’ COVID-19 status.
4. All HCW have been equipped with personal thermometers to measure body temperature twice daily
5. Cleaners wore approved PPE during the cleaning activity. All plastic coverings were removed carefully to avoid accidental aerosolization of particles, and then placed in biohazard bags. Any unused disposable within the room was discarded; hence, only essential items had to be brought and available into the procedure room.
6. IRs and technologists were instructed to properly and safely handle and decontaminate IR equipment.
7. Strict decontamination measures in IR theaters, floor cleaning with liquid disinfectant, and replacement of all medical sheets, were taken after each procedure.

IR direct exposure to the SARS-CoV-2 virus include vascular access (central venous lines, distal perfusion catheters for extracorporeal membrane oxygenation) and drainage of pleural or peritoneal fluid collections. However, virtually any interventional procedure may be required in an infected or suspected patient.

Potentially acute situations may arise during the procedure (i.e. collapse during embolization of acute bleeds requiring cardiopulmonary resuscitation). It is important to anticipate the critical event and plan dedicated protocols for various scenarios in advance.

Anyone who was in any affected area or exposed to COVID-19 patients without proper PPE was quarantined for at least 14 days and tested by PCR on oropharyngeal swabs before re-admission.

## Anti-COVID-19 task force

The established COVID-19 task force under the coordination of Dr. Miscio, the Medical Director of the Institute, elaborated a strategic prevention plan to (1) limit the access of family members at the Institute admission as well as at the daily appointment/gathering with their relatives; (2) prevent the admission of COVID-19-positive patients with an evolving triage: initially performed by thermal monitoring and interview on the health conditions of the previous 10 days (including cough and respiratory symptoms), subsequently by IgM/IgG quick serology testing and finally viral PCR detection on oro-nasopharyngeal swabs; (3) weekly serological and swab monitoring of all health workers in activity, with one third of them relocated at home for remote smart working.

## Triage for patients access

A rigorous triage procedure has been elaborated since March 10th following the establishment of the COVI-19 Task Force. The aim was to prevent transmission of SARS-CoV-2 intercepting, in accordance to international organization, subjects who may have been infected by the virus [79, 80].

Triage procedures were implemented with the aim of maintaining standard hospital access rates for cancer patients in need of active treatment and reducing delays for those in need of a diagnostic intervention. The on-site patient workflow was first regulated by establishing two triage checkpoints located at the hospital entry points, where dedicated nursing staff were assigned to care for patients and all individuals (including caregivers) walking to the hospital. In particular, the triage stations were at the entrance to the medical department building.

A nursing staff, designated to have physical contact with patients and caregivers, was selected and adequately trained for the most appropriate use of PPE, administering specific pre-triage questionnaires and collecting / evaluating physical parameters. A specific triage procedure was established, which was continuously updated and improved (10 revisions to date).

Procedure involved:

1. Temperature measurement by infrared thermometers and verification by tympanic thermometers;
2. Assessment of blood oxygen saturations for patients with clear breathing difficulties, cyanosis, confusional state and all those in wheelchairs;
3. Administration of a questionnaire to identify possible previous contacts with COVID-19 patients or with swab- positive subjects or patients with breathing symptoms and/or fever in the previous days;
4. Delivery of facial masks to patients, with not suitable mask or even without any mask;
5. Information on the need of hand hygiene and correct use of gloves. Several hand hygiene stations have been installed in the triage area, the waiting areas and all departments;
6. Entry restriction for caregivers and accompanying family members.

High-risk patients (i.e. with temperature ≥ 37.5 °C, blood oxygen saturation ≤ 90%, at least one sign or symptom of respiratory distress, or declaration of contacts with swab-positive to SARS-CoV-2 individuals) were immediately isolated from patient workflow and evaluated by the reference physician for differential diagnosis with cancer-related symptoms.

In addition to hospital triage, physicians were alerted to arrange a pre-triage telephone call to patients to verify their health status.

**Fig. 7:**
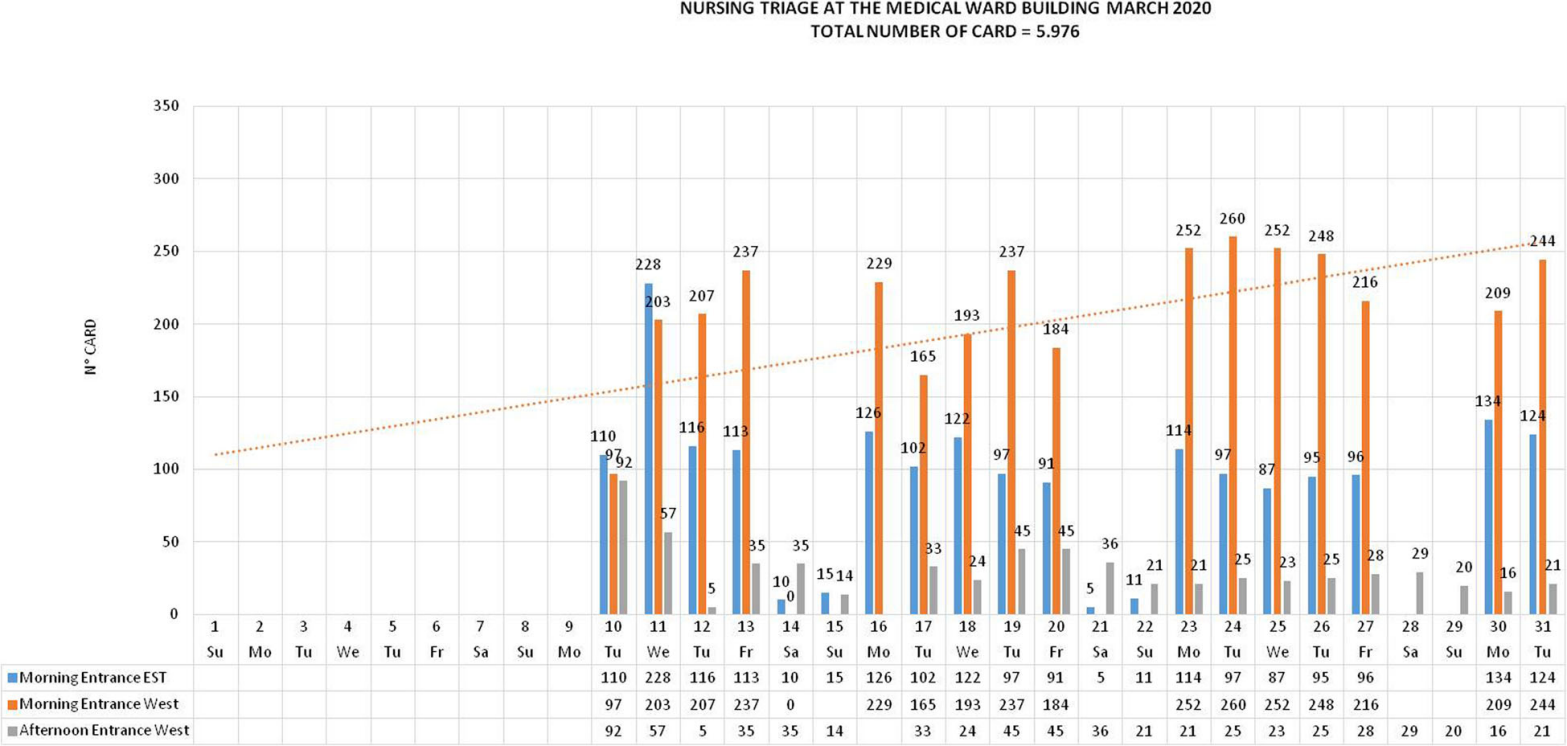
Number of nursing triage performed at Medical ward building in March 2020

**Fig. 8:**
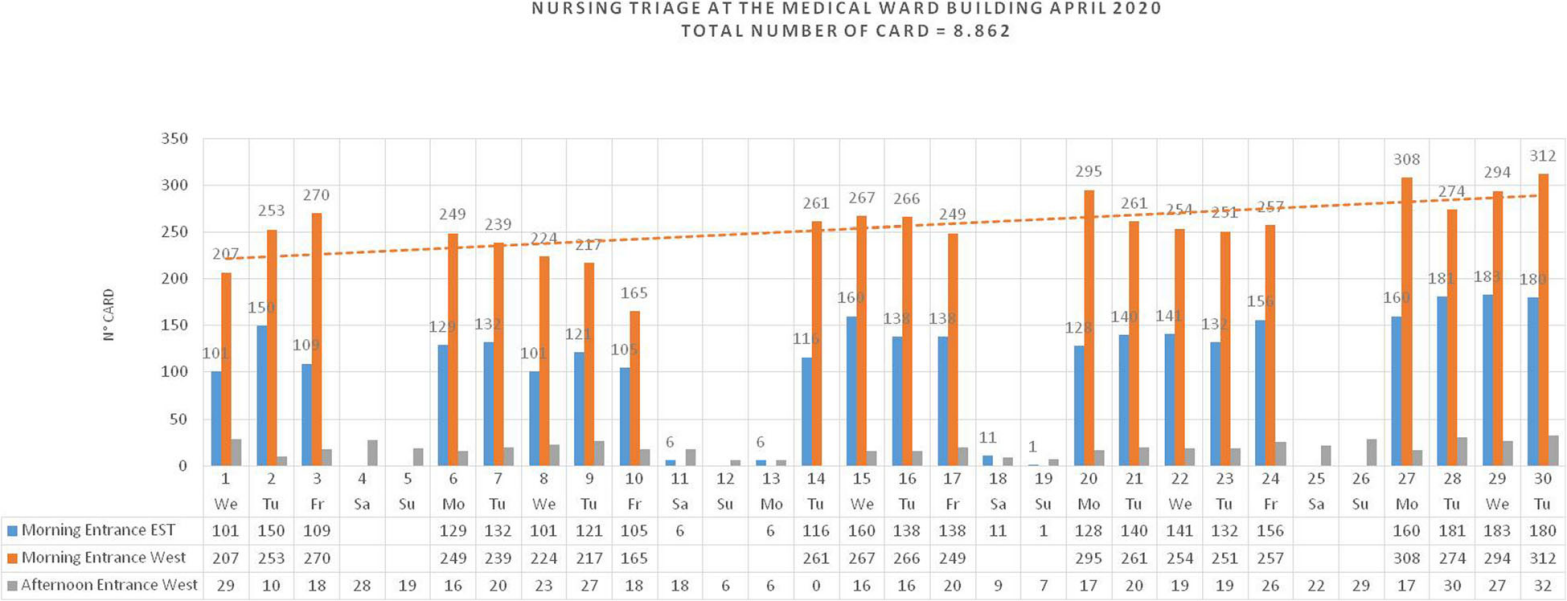
Number of nursing triage performed at Medical ward building in April 2020

**Fig. 9:**
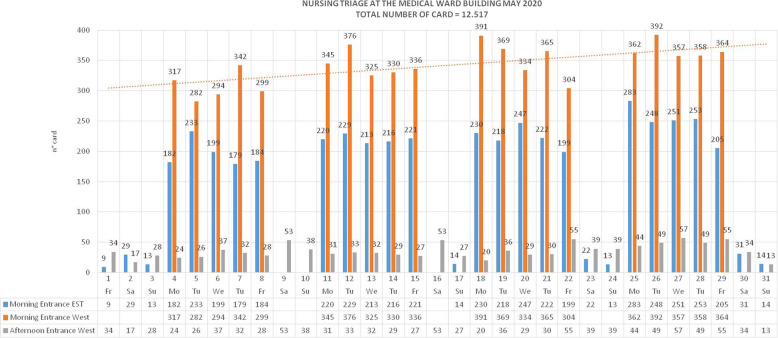
Number of nursing triage performed at Medical ward building in May 2020

**Fig. 10:**
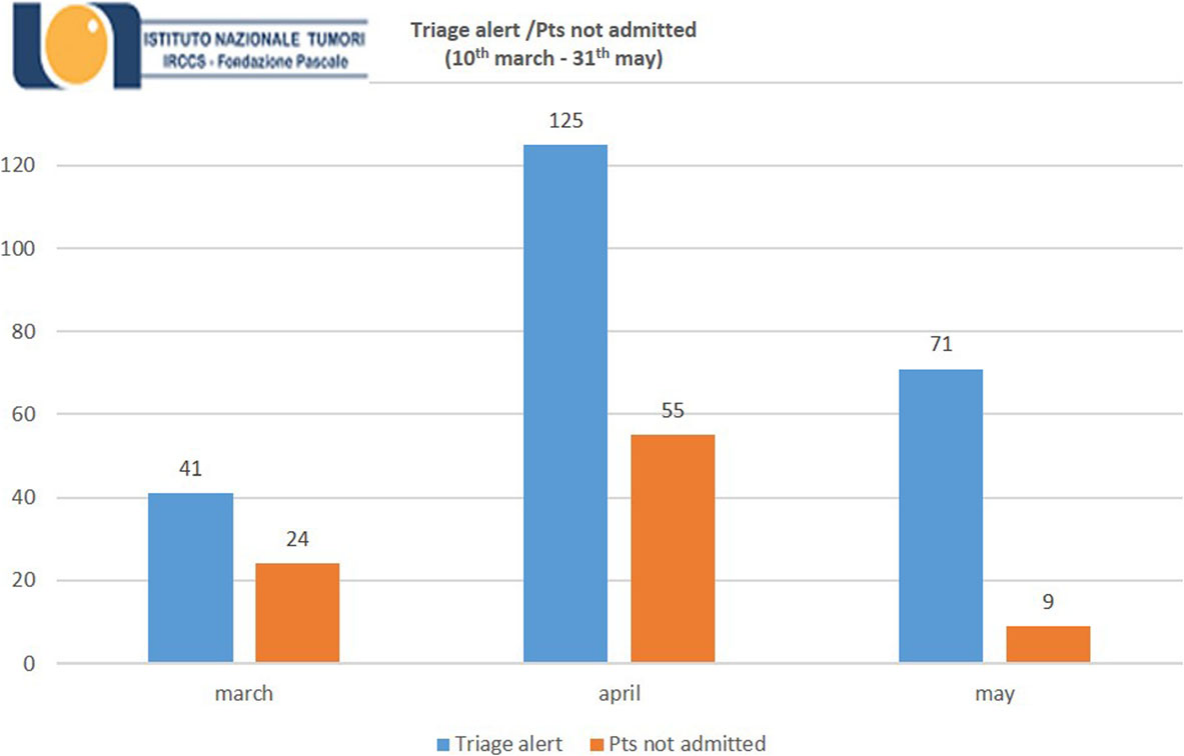
Number of patients not admitted to the oncology wards following a triage for SARS-CoV-2 infection in March, April and May 2020

Initially, 58 clinical nurses in addition to several nursing supervisors/managers guaranteed the activities of the two triage points at the medical ward building, which includes the DH Chemio for over 1300 h. 5976 triage cards were compiled in the 21 days of March (Fig. 7) The total number of nurses involved in the triage raised to 90 for a total number of 2427 h of activity in April. The triage cards with all patient details progressively increased (Figs. 8 and 9). As result, in March 41 patients were identified with minor symptoms and following further investigations (including the reference physician evaluation) 24 patients were not admitted. In April 125 patients were identified at the triage station and 55 of them were not admitted. Finally, in May 71- patients were identified and 9 were not admitted (Fig. 10).

**Fig. 11:**
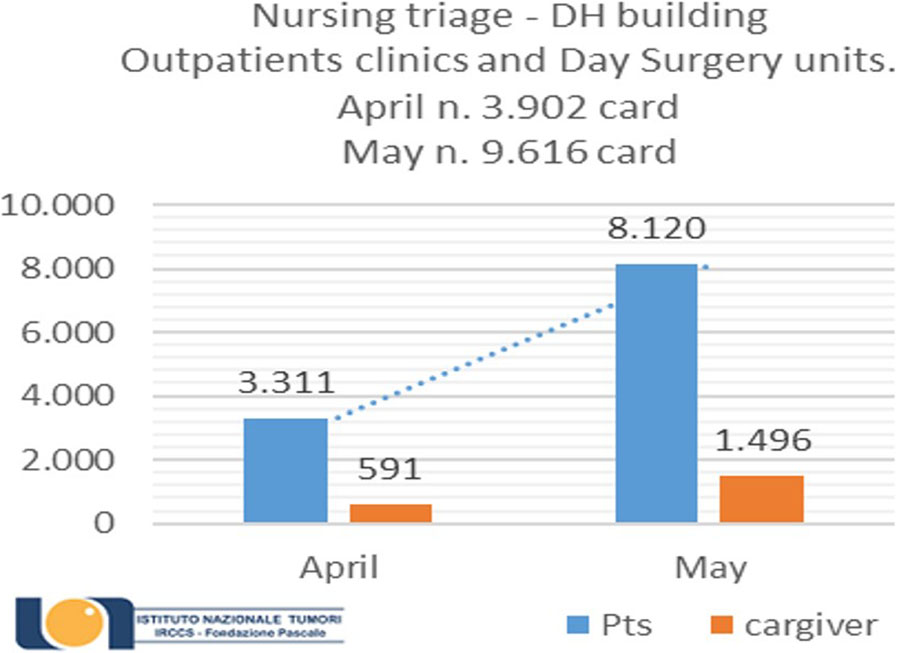
Number of patients and caregivers attending the outpatients clinics and the Day Surgery units in April and May 2020

At the triage points, the temperature monitoring was also performed for all INT-Pascale personnel and outsourced operators permanently or sporadically present in the Institute [595 in March, 564 in April and 1952 in May]. Since April, other triage stations have been established at the DH building, which include outpatient clinics and Day Surgery units with dedicated desks (Fig. 11).

Supervisor nurses collaborated in the development of operating instructions / procedures and were involved in the following operators’ training on: 1) dressing / undressing, hand hygiene, correct use of PPE and in particular of FFP2 masks and code of conduct according to the recommendations of ANIPIO and national directives, for the triage nurses [81, 82]; 2) dressing / undressing, hand hygiene, insulation standards, correct use of PPE and in particular of FFP2 masks for wards nurses and UAP; 3) pharyngeal swab sampling and handling for dedicated nurses, to appropriately follow the operating instruction for the execution of the COVID-19 nasopharyngeal swabs and transfer to the clinical laboratory [83]; 4) execution of rapid SARS-CoV-2 IgM and IgG test performed since 15th of May by ward and Day Surgery nurses. When “suspect cases” were identified the current, updated internal procedures were reviewed for appropriate implementation.

## Medics monitoring

Monitoring the incidence of the SARS-CoV-2 infection among the health workers at the INT-Pascale, has been a major target of the institute, considering the high infection rate among the health workers of the Northern Italy Hospitals and their possible role as asymptomatic spreaders.

According to the Recommendations for the surveillance of healthcare personnel - new coronavirus (SARS – CoV-2) of Campania Region COVID Crisi Unit [84] at the IRCCS National Cancer Institute “G. Pascale” of Naples operational surveillance procedures have been elaborated and implemented for all healthcare, professional, technical and administrative staff workers.

Particular attention was paid to health personnel who have had “close contact” with a subject tested positive for the SARS-CoV-2 without the use of PPE. All this in a more extensive perspective than Ministerial and Regional regulations, which provided for close and unprotected contacts with subjects affected by COVID-19, in order to further guarantee the protection of cancer patients admitted at the Institute and staff, who could have been unknowingly a vehicle of the infection.

Specifically, the healthcare professional, who recognized himself in the definitions of “close contact” of the confirmed SARS-CoV-2 case, had to fill in two electronic forms to describe: the incident (the occasion of the close contact) and the daily self-surveillance report of any symptoms related to the SARS COV-2 infection in the following 14 days. These reports have been analyzed within COVID-19 Crisis Unit working group, established at the Institute on March 10th, consisting of the Hospital Medical Director, the Occupational physician, the Director of the Epidemiology and Biostatistics Unit, the Infectious disease physician, the Director of Laboratory Medicine Unit and the Director of Molecular Biology and Viral Oncology Unit.

The working group has met daily to draw up the list of workers to be tested by the PCR-based molecular diagnostic procedures, which was sent to the Head of the Structure and the Nursing Coordinator of the Operator’s home for further contacts. Both symptomatic (body temperature ≥ 37.5 °C as well as respiratory symptoms as cough and difficulty breathing) and asymptomatic workers, have been sampled with an oro- and nasopharyngeal swab. In case of PCR-positive for SARS-CoV-2 sequences, the worker was home quarantined for 14 days, activating contacts with the general practitioner (GP). In case of negative results, symptomatic workers remained at their home with molecular testing reevaluation, while the asymptomatic were promptly readmitted to work, with the use of PPE and surgical mask. Self-monitoring of their own clinical conditions (daily reported on the monitoring card) along with nasopharyngeal swab every 48 h was continued for a 14-day period after close contact with the molecular confirmed SARS-CoV-2-positive case. Moreover, since May 1st in compliance with “Regional operational protocol for the early identification of infected COVID-19 through the use of rapid tests” [85], epidemiological surveillance of all personnel of the Institute has been performed by rapid serological tests, based on immunochromatographic detection of anti Sars-CoV-2 IgM and IgG. Antibody-positive workers were further tested by nasopharyngeal swabs.

## Patients and care givers Psycological support

COVID-19 pandemic had a strong psychological impact, particularly on cancer patients with underlying anxiety related to their oncological disease. Psychological interventions had to be diversified according to their health conditions. Cancer patients were affected by different diseases and at different stages. Furthermore, they had different personalities and tempers.

Patients experienced a double trauma, distress linked to their illness and to the COVID-19 pandemic. Both patients and family members feared that they would not receive the necessary and adequate care for the emergency situation, the emotional stress experienced by the healthcare professional and the remodulation of follow-ups as well as primary visits.

The forced distance from family members, moreover, at the moment of hospitalization and/or during treatments amplified the sense of anguish and loneliness.

In this alarming condition, in order to contain severe psychological distress and to reduce risk factors, it has been necessary more than ever to ensure psychological support for patients and family members, including multimedia support. Finally, in order to improve patient care and quality of life, it has been also essential to guarantee support for healthcare personnel. In this regard, a listening desk for health professionals has been activated and operated daily at the Psychology Unit.

## Remote home monitoring planning

A further relevant step taken by the management during the 3 months (March–May) of COVID-19 has been to accelerate the planning of home remote monitoring already activated for oncological diseases with high monitoring demand (i.e. breast and colon cancers). In this respect two major approaches were taken: a) a home health care platform, able to collect by remote device and patients’ daily reports parameters (i.e. temperature, blood pressure, gastrointestinal and respiratory symptoms) needed for appropriate home monitoring; b) an intensive health care platform (IHCP), activated to perform a continuous h24 monitoring and analysis of vital sign (including ECG, oxygen saturation, cardiac and respiratory rate) for rapid intervention.

Besides the two platforms, monitored by two physicians each, few devices were taken into consideration: Bluetooth connected conventional medical devices, as well as wearable devices including medical sensor patch and wearable smart ScanWatches. Such advanced remote strategies have been introduced and implemented also in clinical trials by several CRO (Contract Research Organization) for decentralized remote monitoring of treatment outcomes (Electronic Patient Reported Outcomes -ePROs) and even for Serious Adverse Event (SAE) data collection and notification [86, 87]. In this context IHCP and wearable devices have been planned for COVID-19 targeted phase II clinical trials for repurposing of commercially available molecules (i.e. Favipiravir and Camostat).

## Conclusions

This article represents an Institutional report of the internal reorganization implemented in order to continue the Cancer treatment activity, at the CCC INT-IRCCS “Fondazione Pascale” Cancer Center in Naples, and to protect from COVID-19 infection the hospitalized cancer patients and those attending the outpatient clinics, their caregivers and the Institute health workers. The established COVID-19 crisis units prevented the transmission of the infection to the hospitalized “fragile” cancer patients (particularly onco-hematological patients), immediately intercepting COVID-19 positive patients at the hospital admission, promptly identifying COVID-19 positive patients (*n* = 2), and transferring them to a temporary quarantine section of the semi-intensive care unit, as well as health workers infected outside of the Institute and activation of the mandatory home quarantine procedure. The Institute quick reaction at the mounting epidemics, even in absence of COVID-19 patients, achieved highly relevant research results and in particular highlighted the anaphylactoid component of the Acute Respiratory Distress Syndrome (ARDS) and the possibility of introducing anti-IL-6/IL-6R axis available drugs in particular Tocilizumab during the cytokine storm with drastic reduction of the Intensive Care Units (ICU) occupancy and the following intubation (9–11). This innovative treatment approach led to the elaboration of the Phase II clinical trial TOCIVID-19 [EudraCT number 2020–001110-38 – ClinicaltTrials.gov NCT04317092] approved by AIFA (the Italian Drug Agency) on March 17th [88], representing the first non-profit anti-COVID-19 Italian clinical trial [89]. A similar study with a different anti-IL6R mAb (Sarilumab, distributed by Regeneron-Sanofi) was announced by the company on March 16th [90] and by FDA on March 19th [91]. Since then, several research activities, particularly in the translational area, were coordinated by the Scientific Director, Dr. Gerardo Botti, with participation at the COVID-19 research programs launched and funded by the Campania Regional Government and by the Italian Ministry of Health. In this context other molecules were identified and their repurposing studied in particular Sofosbuvir [92, 93] along with Favipiravir and Camostat. Moreover, under the auspices of the Director General, Dr. Attilio Bianchi, who strongly supported long-term post-treatment and inter-treatment cancer health programs, an innovative home-based remote monitoring was elaborated and implemented with development of digital device and an appropriate IT platform for h24 collection of COVID-related vital date (including body temperature, SpO_2_ and ECG). This strategy allowed the home monitoring of COVID-19 positive subjects at risk of further progression to severe respiratory distress.

In conclusion the IRCCS Fondazione Pascale in agreement with the Regione Campania political strategy, contributed at scientific as well as at clinical care level to provide oncological and surgical care to the Campania Cancer patients and to contain SARS-CoV-2 transmission among cancer patients and Health workers elaborating an unprecedented knowledge management and innovation approach, which although difficult to implement in Health Care Institutions is crucial to provide the best possible healthcare, achieve operational excellence, and foster innovation [94–96].

## Data Availability

All relevant data are included in the article.

## Abbreviations

ACE: Angiotensin-converting enzyme
ACE2: Angiotensin-converting enzyme type 2
ARDS: Acute Respiratory Distress Syndrome
BRAFi/MEK inhibitors: drugs able to inhibit B-Raf kinase and the MEK1/2 pathway
CCC: Comprehensive Cancer Center
COVID-19: CoronaVirus Infectious Disease of 2019
CT: Computer Tomography
DCIS: Ductal carcinoma in situ (DCIS)
DH building: Day Hospital building
EBC: Early Breast Cancer
ECG: ElectroCardioGram
ENT doctor: Ear, Nose, and Throat doctor or *otolaryngologist*
ENT examination: Ear, Nose, and Throat examination
FFP2 mask: Filtering FacePiece 2 EN 149 filtering 94% of airborne particles (including coronavirus) equivalent to N95 USA and KN95 China.
G-CSF: Granulocyte Colony-Stimulating Factor
GCP: Good Clinical Practice
GEP-NET: Gastroenteropancreatic NeuroEndocrine Tumors
GOM: Gruppo Oncologico Multidisciplinare (Multidisciplinary Oncological Group)
HCW: Health Care Workers
HEPA filtration: High-Efficiency Particulate-Absorbing filtration
HER2: Human Epidermal Growth Factor Receptor 2
ICU: Intensive Care Unit
IMP: INVESTIGATIONAL MEDICINAL PRODUCTS
IHCP: Intensive Health Care Platform
INT: Istituto Nazionale Tumori (National Cancer Institute) – IRCCS “Fondazione Pascale”, Napoli
IRCCS: Istituto di Ricerca e Cura a Carattere Scientifico (Comprehensive Research and Treatment Cancer Institute)
LHRH-analogue: Luteinizing Hormone-Releasing Hormone (LHRH) agonist
MBC: Metastatic Breast Cancer
m-TOR: mammalian Target of Rapamycin
OS, PFS or QoL: Overall Survival; Progression-free Survival; Quality of Life
PDAC: Pancreatic Ductal AdenoCarcinoma
PAPR masks: Powered Air Respiratory Protection masks
POC: Point of Care
POCUS: Point-Of-Care UltraSound
PPE: Personal Protective Equipment
PRO questionnaires: Patient-Reported Outcome (*PRO*) *questionnaires*
*PSD*: *Personal Safety Device*
*RT-PCR*: *Real Time – Polymerase Chain Reaction*
*SARS-CoV-2*: Severe Acute Respiratory Syndrome associated to CoronaVirus two of 2019
SpO_2_: Peripheral capillary oxygen saturation
TORS: TransOral Robotic Surgery
UAP: Unlicensed assistive personnel
ULPA Filter: Ultra Low Penetrating Air Filter
AIFA: Agenzia Italiana del FArmaco (Italian Medicines Agency)
AIOM: Associazione Italiana Oncologi Medici (Italian Association of Medical Oncology)
ANIPIO: Società Scientifica Nazionale Infermieri Specialisti del Rischio Infettivo (Italian Society for Professionals in Infection Control
CIPOMO: Collegio Italiano dei Primari Oncologi Medici Ospedalieri (Italian College of Chief Medical Oncologists)
COMU: Collegio degli Oncologi Medici Universitari (Italian College of University Medical Oncologists)
EMA: European Medicines Agency
ESMO: European Society of Medical Oncology
SIAPEC-IACP: Società Italiana di Anatomia Patologica e Citologia Diagnostica-Divisione Italiana della International Academy of Pathology
